# COVID-19 Vaccination in Lower-Middle Income Countries: National Stakeholder Views on Challenges, Barriers, and Potential Solutions

**DOI:** 10.3389/fpubh.2021.709127

**Published:** 2021-08-06

**Authors:** Eunice Twumwaa Tagoe, Nurnabi Sheikh, Alec Morton, Justice Nonvignon, Abdur Razzaque Sarker, Lynn Williams, Itamar Megiddo

**Affiliations:** ^1^Department of Management Science, University of Strathclyde, Glasgow, United Kingdom; ^2^School of Public Health, University of Ghana, Legon, Ghana; ^3^Population Studies Division, Bangladesh Institute of Development Studies (BIDS), Dhaka, Bangladesh; ^4^School of Psychological Sciences & Health, University of Strathclyde, Glasgow, United Kingdom

**Keywords:** COVID-19 vaccination, vaccine delivery, vaccine hesitancy, vaccine provider challenges, stakeholder perspective

## Abstract

The development of COVID-19 vaccines does not imply the end of the global pandemic as now countries have to purchase enough COVID-19 vaccine doses and work towards their successful rollout. Vaccination across the world has progressed slowly in all, but a few high-income countries (HICs) as governments learn how to vaccinate their entire populations amidst a pandemic. Most low- and middle-income countries (LMICs) have been relying on the COVID-19 Vaccines Global Access (COVAX) Facility to obtain vaccines. COVAX aims to provide these countries with enough doses to vaccinate 20% of their populations. LMICs will likely encounter additional barriers and challenges rolling out vaccines compared HICs despite their significant experience from the Expanded Programme on Immunisation (EPI). This study explores potential barriers that will arise during the COVID-19 vaccine rollout in lower-middle-income countries and how to overcome them. We conducted sixteen semi-structured interviews with national-level stakeholders from Ghana and Bangladesh (eight in each country). Stakeholders included policymakers and immunisation programme experts. Data were analysed using a Framework Analysis technique. Stakeholders believed their country could use existing EPI structures for the COVID-19 vaccine rollout despite existing challenges with the EPI and despite its focus on childhood immunisation rather than vaccinating the entire population over a short period of time. Stakeholders suggested increasing confidence in the vaccine through community influencers and by utilising local government accredited institutions such as the Drug Authorities for vaccine approval. Additional strategies they discussed included training more health providers and recruiting volunteers to increase vaccination speed, expanding government budgets for COVID-19 vaccine purchase and delivery, and exploring other financing opportunities to address in-country vaccine shortages. Stakeholders also believed that LMICs may encounter challenges complying with priority lists. Our findings suggest that COVID-19 vaccination is different from previous vaccination programs, and therefore, policymakers have to expand the EPI structure and also take a systematic and collaborative approach to plan and effectively rollout the vaccines.

## Introduction

The development of multiple effective COVID-19 vaccines in less than a year is a remarkable achievement, but successful protection of populations globally will also rely on the availability of vaccine doses and the campaigns countries run to immunise their populations. As the adage says, “vaccines do not save lives; vaccination does.” Several high-income countries (HICs) have made significant headway, with Israel leading the way, vaccinating 50% of its population by the end of February (1). Many low- and middle-income countries (LMICs) are relying on support through the COVAX (COVID-19 Vaccines Global Access) Facility, which is led by the World Health Organisation (WHO), Gavi, and the Coalition for Epidemic Preparedness Innovations. COVAX has been utilising donor funds in its Advance Market Commitments as it aims to ensure equitable access to COVID-19 vaccines. Vaccination campaigns have begun in a few of these LMICs.

COVID-19 vaccination has been challenging in many HICs. Challenges such as vaccine hesitancy and scarcity of doses have been anticipated, with potential solutions such as mass media interventions and population groups prioritisation proposed (2–5). These interventions may have helped to increase uptake and efficiency of vaccination rollout, but they have not entirely resolved all issues. Further administrative challenges have arisen as HICs began vaccinating, and new solutions to overcome supply barriers, such as increasing the spacing between vaccine doses, have been proposed (2, 6). Undoubtedly, governments rolling out vaccines will continue to face new issues as they learn how to vaccinate their entire (or most of their) population during a pandemic.

LMICs may face greater and additional challenges compared to HICs despite their significant experience from the Expanded Programme on Immunisation (EPI), which launched in 1974. LMIC governments have made significant improvements in immunising their populations. Nonetheless, they face challenges due to vaccine hesitancy (7), low resource availability (8), poor roads to transport vaccines (9), inadequate cold-chain and storage (7), lack of coordination with a significant private healthcare sector (10), and limited funds for surveillance (11) among other issues. Further, most EPI vaccines are for children and are administered at defined intervals during infancy, while COVID-19 vaccination will require vaccinating most of the population as quickly as possible. Though learning from the EPI is important, administering COVID-19 vaccines poses new or larger challenges.

Recent studies have investigated COVID-19 vaccine rollout in LMICs and reported challenges with ensuring LMICs have adequate COVID-19 vaccines to achieve herd immunity. HICs have bought most of the vaccines, including those yet to be produced (12, 13). Burgess et al. (2021) and Figueroa et al. (2021) emphasised LMICs cannot afford the price of COVID-19 vaccines for their population (14, 15). Research has highlighted that insufficient vaccine manufacturing is a significant hurdle (16) India and South Africa have proposed manufacturers in HICs relinquish intellectual property rights associated with COVID-19 vaccines and therapeutics to increase manufacturing and access in LMICs; however, the pharmaceutical industry disagrees and describes the idea as a discouraging innovation (17).

Even if countries have sufficient COVID-19 vaccine doses, distribution and monitoring are likely to slow down rollout. Alam et al. (2021) found the most important challenges for vaccination include lack of coordination among local organizations, lack of vaccination monitoring authorities, and inadequate monitoring and controlling of vaccine temperatures (16). Similarly, Mesa-Vieira et al. (2021) noted inefficiency in maintaining vaccination target group, lack of surveillance and monitoring, logistical problems, vaccine hesitancy, miscommunication among the communities, and a weak health system as hurdles for the LMICs (18). Other studies also identified weak political commitment, poor law enforcement (14, 15, 19).

Lastly, vaccine acceptance and hesitancy remain a significant issue, though studies have found mixed results on vaccine hesitancy in LMICs compared to HICs. Bhopal and Nielsen et al. (2021) noted hesitancy as an important barrier in LMICs (19). Arce et al. (2021), in a study involving ten LMICs, found that individuals in these countries are more likely to accept COVID-19 vaccines (on average 80%) than ones in the United States (65%) or Russia (30%) (20). A willingness to vaccinate study conducted by Abedin et al. (2021) in all eight regions in Bangladesh among 3,646 participants based on a household survey found that 74.6% of respondents agreed to accept a free, safe, and effective vaccine, while 46.5% were willing to pay a small fee to be vaccinated (21). Vaccine refusal and hesitancy were higher among the rural, semi-urban, slum, elderly, and low-educated populations (21). In Ghana, a study conducted among the 2,734 people in all sixteen regions of the country found that 82.8% of respondents were willing to take a COVID-19 vaccine, while 9.7% were hesitant (22). The reasons for hesitancy vary, with the most common reasons being unpleasant side effects, vaccination disinformation and lack of information, and lack of trust in the government and pharmaceutical companies (22). In a study conducted in five LMICs, including Bangladesh, vaccine acceptance was linked to social norms, perceived risk of COVID-19, belief in vaccine effectiveness, perceived safety of vaccines, and presumed access to vaccines (23).

The aim of this study is to utilise the experience and views of national stakeholders in lower-middle-income countries to explore these and other potential issues that will arise during the COVID-19 vaccine rollout in LMICs, as well as how to overcome them. Specifically, we investigate rolling out the vaccine in two LMICs, Bangladesh and Ghana. To our knowledge, this is the first study to examine COVID-19 vaccination challenges in Ghana and Bangladesh from the perspective of national stakeholders. We discuss potential barriers and challenges along the path of immunising the population: planning, financing of delivery, infrastructure and supply-chains, human resources for delivery, the population willingness to vaccinate, and monitoring and evaluation of delivery.

## Methods and Materials

### Study Design and Setting

This study was conducted in Ghana and Bangladesh to explore commonalities and differences in COVID-19 vaccination challenges and planning among countries with similar World Bank classifications but from different regions. Both countries offer free immunisation through the EPI supported by Gavi, which primarily covers infants and children. Interviews with national stakeholders, including policymakers and immunisation programme experts, were semi-structured and followed a topic guide. The guide ensured all important steps for getting individuals vaccinated were discussed while allowing interviewees to provide additional open-ended information. Three authors developed the topic guide from findings of a narrative literature review on vaccination in LMICs conducted for this study. Topics covered included supply-side and demand-side barriers and potential strategies to overcome them. All the study authors reviewed and validated the topic guide for appropriateness and comprehensiveness of the topics covered. Interviews were conducted between October and November 2020, before COVID-19 vaccination had commenced in these countries. All interviews were conducted using the same topic guide.

### Interviewees and Recruitment

We sought to interview national stakeholders, defined as individuals with interest and expertise in COVID-19 vaccination rollout in their country. In particular, we identified professionals and academics involved in health care policy and delivery. We used a purposive sampling approach to select twenty-eight stakeholders (13 from Ghana and 15 from Bangladesh) from the Ghana Health Service and the Ghana Ministry of Health website, the Bangladesh Ministry of Health and Family Welfare Division, University of Dhaka, Bangladesh and International Centre for Diarrhoeal Disease Research, Bangladesh. Using purposeful sampling, the research team carefully identified and selected a reasonable representation of potential interviewees directly involved in national planning of vaccination rollout in both countries. The selected national stakeholders were invited via an email invitation to participate in the interviews, and those that agreed to participate were asked to sign consent forms. The invited stakeholders have all been engaged in immunisation activities and have had experience or knowledge on the immunisation process but come from unique perspectives in order to get diversified information. The invited interviewees included officials from the EPI and the Ministry of Health, academics, and policymakers. We used snowball sampling to augment the purposive sampling such that interviewees were asked to recommend similar national-level stakeholders for an interview (24). Sixteen stakeholders (fourteen initially invited and two referred/recommended) accepted the invitations and were interviewed. Eight personal interviews were conducted in each country, Ghana and Bangladesh, after which no further interviews were conducted because of data saturation, i.e., receiving similar responses for the same questions. Table 1 indicates the institution and role of interviewees.

**Table 1:** Characteristics of study participants.

**Participant number**	**Country**	**Institution/Designation**	**Role and Expertise**
1B	Bangladesh	Ministry of Health and Family Welfare, Bangladesh	Immunisation service delivery
2B	Bangladesh	Ministry of Health and Family Welfare, Bangladesh	Immunisation service delivery
3B	Bangladesh	Ministry of Health and Family Welfare, Bangladesh	Research, planning and development
4B	Bangladesh	Ministry of Health and Family Welfare, Bangladesh	Health economics research, planning and development
5B	Bangladesh	Ministry of Health and Family Welfare, Bangladesh	Health economics research, planning and development
6B	Bangladesh	University of Dhaka, Bangladesh	Health financing and policy research
7B	Bangladesh	Ministry of Health and Family Welfare, Bangladesh	Research, planning and development
8B	Bangladesh	International Centre for Diarrhoeal Disease Research, Bangladesh	Vaccine research, planning and development
9G	Ghana	Ghana Health Service	Health administration and support service
10G	Ghana	Ghana Health Service	Health research and development
11G	Ghana	Ghana Health Service	Clinical and engineering services
12G	Ghana	Ghana Ministry of Health	Health policy planning, monitoring and evaluation
13G	Ghana	Presidential office	Health policy and programmes
14G	Ghana	Ghana Health Service	Procurement of goods and services
15G	Ghana	Ghana Health Service	Health policy planning, monitoring and evaluation
16G	Ghana	Ghana Health Service	Public health

### Data Collection

Study interviews were conducted via zoom and telephone calls. Interviewers asked participants to describe their role and expertise in relation to health policy decision making and vaccine delivery. The topics covered in the topic guide were based on a prior literature review. They included barriers due to vaccine hesitancy, funding, planning capacity, infrastructure, transportation, human resource, and monitoring, as well as strategies to overcome these barriers. Interviewers also asked about further topics not covered. The topic guide is available in the supplementary documents.

Ethics approval for the study was granted by the research ethics committees at the University of Strathclyde and the Ghana Health Service. Ethics approval obtained from the University of Strathclyde was sufficient to permit interviews in Bangladesh, and therefore, no further approval was required in Bangladesh. Prior to the interviews, interviewers explained the study purpose, provided participant information forms, and requested individual consent from the interviewees. Interviews lasted 30–40 minutes on average and were recorded after approval from the interviewees. The interviewers have had prior experience in qualitative interviews, and the research team extensively discussed the approach for the interviews. Interviews were conducted in English in Ghana and Bangla in Bangladesh. They were transcribed and translated into English (if needed). Transcripts were securely stored in a file with relevant information, including interview date, time, etc. The assigned team members checked the accuracy of the transcription process for two randomly selected interviews in each country.

### Data Analysis

Data analysis was performed using the Framework analysis method, which comprises five stages: (i) familiarisation, (ii) identifying a thematic framework, (iii) indexing, (iv) charting, and (v) mapping and interpretation (25, 26). Two researchers thoroughly read the entire interview transcripts they were analysing (familiarisation).

The thematic framework was developed using both deductive and inductive codes. The deductive codes were identified through a narrative literature review conducted for this study and grouped under demand- and supply-side barriers. The researchers applied the collection of deductive codes to four randomly selected transcripts. Both researchers coded the same transcripts. The collection of deductive codes was revised to include inductive codes, emerging from the first four coded interview transcripts. The revised framework had seven categories: three demand-side and four supply-side barriers and strategies (reported in section findings). The entire research team agreed on the thematic framework.

The revised framework was applied to the remaining twelve interview transcripts (indexing), and all coded data were summarised and charted into matrices to allow interpretation. A matrix was developed for each broad category. Each matrix row represented an interviewee, and each column represented a code. Cells contained summarised data. The research team then reviewed and discussed the data summarised in the matrices and proceeded to interpret it.

## Findings

This section reports the study results under seven themes (grouped under supply-side and demand-side barriers) identified during the literature review and analysis phase. Table 2 shows a definition of the seven themes. We also report strategies to overcome the barriers and challenges discussed in interviews.

**Table 2:** Definition of themes.

**Category**	**Theme**	**Definition**
**Demand-side barriers**	Vaccine safety concerns	Peoples' beliefs about the COVID-19 vaccine and concerns about adverse events.
	Vaccination service-related barriers	Vaccine consumers' concerns with the timing of vaccination, location of vaccination sites and the financial cost of getting vaccinated.
	Social/religious and culture-related barriers	Matters of complacency, social group resistance and negative social/cultural beliefs about the COVID-19 infection and vaccination
**Supply-side barriers**	Vaccine production and cost-related barriers	Barrier with manufacturing and purchase of vaccines, e.g., limited funds and limited production capacity
	Vaccine distribution and storage-related barriers	In-country cold-chain system capacity and maintenance, e.g., few fridges, erratic power supply, vaccine carriers, cold vans, poor roads, and few maintenance engineers.
	Vaccine delivery and administration-related	Barriers associated with vaccination service provision, e.g., few providers, discipline around priority list, less involvement of private sector, inaccessible places and person, and congested sites
	Vaccination program monitoring and evaluation	Challenges with vaccination program surveillance and data management, e.g., suboptimal data management software, poor internet connectivity, few trained IT health staff and difficulty estimating coverage parameters.

### Demand-Side Barriers

#### Vaccine Safety Concerns

Stakeholders discussed how beliefs of vaccine inefficacy and adverse events following vaccination increase vaccine hesitancy in the public. Concerns of vaccine efficacy emanate from the speed at which COVID-19 vaccines have been developed, which has raised questions on adherence to standard vaccine production processes, particularly in the trial and accreditation phase. A participant explained that “*People will reject uncertified vaccines. If there is going to be any hesitancy, it will come about if we want to administer vaccines that are still in the trial phase.”* (Participant 10G).

Additionally, stakeholders believed that the fear of adverse events following vaccination, including vomiting, headache, and body pains, will be associated with people delaying or rejecting vaccination. An interviewee said, “*There would be some sceptics who would want to watch and see which side effects people who go and take it have*.” (Participant 10G).

Stakeholders from both countries suggested public education and utilising community influencers to create awareness and contradict incorrect information about the virus and vaccination. Electronic and print media are viable platforms to educate the public. One participant said, “*I am of the view that as early as possible, we get the opinion leaders; we need to bring them on board. …, I think we have to make sure that we get traditional leaders, opinion leaders, and other influential persons on board for support with public education.”* (Participant 14G).

Stakeholders in Ghana and Bangladesh reported that national vaccine approval by government accredited institutions, e.g., Drug Authorities, could allay the public's doubts and fear about vaccines, thereby increasing acceptance. According to an interviewee, “*You cannot introduce it just then, so it has to be approved by the regulatory authority of the country. In this case, our authority is Directorate General of Drug Authority (DGDA)”* (Participant 2B). Similarly, a stakeholder from Ghana said that “*aside from approval from international communities, we will need approval from the Food and Drugs Authority.”* (Participant 10G).

#### Vaccination Service-Related Barriers

Interviewees identified vaccination fatigue and the direct and indirect cost of vaccination to consumers as vaccine uptake barriers. Vaccination fatigue may apply if vaccines are administered in multiple doses plus future boosters, implying that consumers may be required to visit vaccination sites more than once to complete vaccination. An interviewee explained the issue of fatigue: “*Some, I think just get tired because the vaccines are now so many so they come after one year and they are eventually giving up even though there are second doses that they need to take.”* (Participant 10G).

Another concern is whether vaccines will be sold or administered free of charge to the final consumer. Study participants in both countries reported that some of the public will undeniably refuse vaccination if required to pay for vaccines. According to one interviewee, “*If vaccines are to be sold to the individual recipient, Ghanaians would not be willing to buy and get vaccinated.”* (Participant 15G). Unaffordable travel costs and time to reach vaccination centres located far from residential places add to the cost barrier and discourage people from getting vaccinated.

Participants did not discuss strategies to address vaccination service-related barriers.

#### Social/Religious and Culture-Related Barriers

Stakeholders said that complacency and group resistance is associated with vaccine rejection. Complacency describes a sense of non-existence of the COVID-19 virus and a belief in being insusceptible to infection. Participants mentioned this perception is common among less affected social groups like the youth “*because COVID-19 has not threatened them so much. Even when infected, they are asymptomatic and unaware'* (Participant 9G). Stakeholders in both countries said that some population groups believe COVID-19 is a disease of the wealthy and specific religious groups. A participant in Bangladesh reported social group resistance among some tribal and religious groups: “*If we talk to some rural people, they will still say that this is not a disease for everyone; rather, it is a disease of wealthy people, urban people and. even initially there was a perception that it is a disease that only affects the non-Muslims. If people think in this way, then we might have to face … great challenges in rural areas*.” (Participant 6B).

Stakeholders proposed using community influencers and volunteers and public education, similar to strategies to address vaccine safety concerns, complacency, negative religious beliefs, and group resistance. An interviewee said, “*we try to use opinion leaders, civil society organisations. We work together with chiefs, especially chiefs and queen mothers and church leaders, mosque leaders…”* (Participant 13G).

### Supply-Side Barriers

#### Vaccine Production and Cost-Related Barriers

The majority of stakeholders emphasised issues with purchasing sufficient COVID-19 vaccine doses. The government's insufficient funds to purchase vaccines, decreasing donor funds, and insufficient vaccine availability are hurdles LMICs encounter in vaccination programmes. The stakeholders believed that acquiring COVID-19 vaccines will be more challenging than other vaccines considering all countries demand COVID-19 vaccines. Policymakers in both countries discussed planning underway to meet the financial cost of vaccine delivery and to ensure the timely arrival of vaccines in their countries. One stakeholder explained that participating in the COVAX facility “*means you are in co-finance. You do not have to pay all the money as other countries*” (Participant 2B).

Stakeholders in Ghana discussed how co-payment mechanisms—which share the cost of vaccines and their delivery between the government and a donor—have led to vaccine shortages in previous vaccination programmes and could occur in COVID-19 vaccination. Participants recounted instances when “*government delays counterpart payment”* (Participant 9G) resulted in national vaccine shortage and threats from donors to discontinue financial support. Participants in Ghana emphasised that the gradual withdrawal of Gavi's funding increases the financial burden on the government, a burden the government will not necessarily be able to meet. A participant explained that the “*vaccine delivery system in Ghana is heavily donor-dependent, and now, donors are pulling out. Without donor support, the government is incapable of funding the purchase and delivery of vaccines … to immunise the whole country.”* (Participant 10G).

Participants discussed the timing of acquiring COVID-19 vaccines, suggesting they will not be readily available in sufficient quantities when manufacturing starts, but rather, they will arrive in batches. An interviewee said, “[getting sufficient COVID-19 vaccines] *is not possible as we are not getting* [enough] *vaccines at the same time”* (Participant 1B). Another respondent said, “*the first few vaccines that will come out, I am sure they have already been bought”* [by HICs]. (Participant 13G).

#### Vaccine Distribution and Storage-Related Barriers

Stakeholders consistently reported barriers associated with the logistics required to store and transport vaccines appropriately. These barriers include too few fridges, inadequate ice packs and vaccine carriers, and unreliable power supply. Vaccine refrigerator storage seems manageable for EPI vaccine distribution; however, units often break down, and their geographic distribution is inequitable. Further, participants said that health systems need more optimal fridges to store COVID-19 vaccines, especially in primary facilities and rural communities. An interviewee explained the situation: “*We do not have a good number of cold-chain facilities; those that we have, they are not working optimally. There are some of them, honestly, sometimes we miss the appropriate temperature, so some of the vaccines injected are just water, the vaccine loses its potency”* (Participant 14G).

Stakeholders in Ghana reported a shortage of trained biomedical engineers within the government health system to maintain fridges and cold vans. According to participants, the government lacks funds to effectively maintain cold-chain equipment, leaving fridges to “*run without prior preventive maintenance repairs”* (Participant 13G). Another interviewee also said, “*There are few biomedical engineers and technicians responsible for the maintenance of fridges. We are limited in number. Sometimes we train [them], and we lose them to private companies. The challenge … is providing resources for them to visit facilities twice a year and make sure that planned preventive maintenance is on schedule*” (Participant 11G).

A majority of the participants reported that erratic power supply contributes to fridges' suboptimal functioning, threatening vaccine potency. Sudden and frequent power outages described as “*dumsor”*—meaning “power off power on”—results from technical challenges and unpaid electricity bills. According to one interviewee, generators form an alternative power supply, “*however, these generators run on diesel which facilities have no funds to buy*” (Participant 11G).

According to interviewees, too few cold vans, vaccine carriers, and ice packs inhibit the smooth transport of vaccines from national cold rooms to communities. Inadequate vaccine carriers and ice packs mean vaccines may not be kept at the appropriate temperature. Also, providers can carry only a few vaccines from the dispatching facility to the community and then return countless times for a refill, making their work tedious.

Stakeholders explained that poor road networks continue to undermine vaccine transportation. Vaccines are transported on motorbikes due to the deplorable state of roads, and maintaining appropriate vaccine temperature on a motorbike is challenging. Some places require health providers to cross a river. The service provides boats, but these boats and motorbikes are not readily accessible, unfuelled, and unmaintained. Participants describe how “*floods decimate roads networks and prevent smooth transportation; no one goes, and no one comes, and vaccination is delayed in such areas*.” (Participant 9G).

#### Vaccine Delivery and Administration-Related Barriers

An interesting sub-theme that emerged in this study is stakeholders' perception that COVID-19 vaccination will be similar to other vaccination programmes. Stakeholders in both countries suggested that the delivery and administration systems are already set up for EPI and that COVID-19 vaccination can utilise this system. Nevertheless, interviewees from both countries discussed challenges with existing EPI structures. A stakeholder explained that “*the system is quite robust and properly managed, so introducing a new vaccine would not require building another system for its administration. It is just a matter of increasing the scope of the EPIs capacities as it exists now….”* (Participant 11G). Another stakeholder expressed that they “*would not anticipate any problem because we have a very good EPI system which is countrywide, headquartered in Accra and goes through all the stages of the health system from the national, to the regional, to the district and the subdistrict.”* (Participant 10G).

Nonetheless, stakeholders discussed capacity issues due to too few trained health providers to administer vaccines. One participant explained that the system is “*already suffering from [an] inadequate [number of] EPI staff. Now that we are considering to cover the entire population, manpower will be a very challenging issue to handle.”* (Participant 5B). Another participant argued that “*from the human resource [perspective], I don't think that we're in a state for handling this massive COVID-19 vaccination program efficiently.”* (Participant 6B).

Stakeholders explained that volunteers can be recruited to help community health nurses administer orally-ingested vaccines, but injecting vaccines requires formal training. An interviewee explained that “*If the vaccine is injectable, we cannot bring anyone to inject, and that will put pressure on the health staff. I do not think there will be enough providers because even though we have nurses, not all are community health nurses to be able to go out and immunise. There will definitely be pressure on the health staff.”* (Participant 9G).

According to participants, the inequitable distribution of health providers is a barrier to vaccination rollout. A participant described that “*There are some areas where we have more than necessary. There are some areas where it is very difficult. People don't want to go to areas where it is very difficult; amenities are not there.”* (Participant 13G). Moreover, stakeholders reported a “*perception … that the people at the top, at the central or the regional level, are the ones enjoying … COVID-19 resources. … it affects the commitment and enthusiasm of the service providers at the lower level.”* (Participant 14G).

To address the shortage of health providers to administer vaccines, stakeholders proposed supplementing the workforce of community health nurses by recruiting additional health workers with knowledge and experience, such as pharmacists, laboratory technicians, and nurses. According to a participant, “*if it is in injectable form, then we don't have any other options left than to recruit people such as healthcare workers from [a] scientific background.”* (Participant 2B).

Stakeholders also noted the low involvement of the private sector as a barrier to scaling up vaccination. Using human and material resources from the private sector would alleviate the pressure on public health facilities and health workers. Participant 5 pointed out that “*Another big challenge can be the private sector of our country. When the COVID-19 virus was spread in our country, the private sector did not come forward during this situation; [the] Bangladesh government had to face a lot of troubles.”* (Participant 5B).

Geographically inaccessible communities and persons were discussed as barriers to vaccination programmes. Labelled as “*hard-to-reach areas*” (Participant 9G), participants described inaccessible communities on islands and mountains and in valleys, slums and places that are cut-off during the rainy season. Travelling to such places is difficult and takes a long time. One stakeholder explained that “*The country is not uniform in terms of development. The hard-to-reach areas, areas which are cut off during the rainy season and areas where it is very difficult to reach unless they travel for about 5 hours before you reach.”* (Participant 13G).

Participants in Ghana described a medical drone delivery service that was recently introduced and could help overcome the lack of access to hard-to-reach areas. The service, which is being scaled up, aims to bring vaccines close to residential areas and help reduce the stress and cost of travelling far distances to vaccination sites. An interviewee identified this in a statement, “we *have added another level to ensure that vaccines' delivery in the cold-chain is kept at the optimal temperature through the medical drone delivery system. We have it in four areas, serving about 2,000 facilities. We hope to have it in four more distribution centres to serve about 95% of our facilities. Once we finish that one next year, then it means at least the hard-to-reach areas … will not be that much of a problem now …*.” (Participant 13G).

Ghanaian stakeholders also discussed “camp-out” to overcome the barrier of “hard-to-reach” communities. An interviewee described “camp-out” as “*we have a period … a whole service is rendered to some particular people at a point in time. So, we move service providers to those islands and stay there for some days to complete immunisation before returning to their post*.” (Participant 10G).

Even so, migrating populations and individuals with illegal residential status are likely to be missed. Vaccination teams visiting communities miss such people because they are either away for a few days or are afraid to expose their legal status. One interviewee suggested that “*Floating people, day labourers etc. might be a bit difficult to gather under the vaccine coverage.”* (Participant 5B). Another interviewee said that “*The missing part of Bangladesh can be because of the geographical reasons and because of the floating people who live here and there; for example, people who are informally working in the capital city*.” (Participant 6B).

Stakeholders in both countries were concerned with the challenge of generating and adhering to a vaccination priority list. They know that their initial vaccine supply will not sufficiently meet demand, resulting in in-country vaccine rationing. The majority of stakeholders believe that in practise, adherence to a priority list will be low. People may use power and influence to get vaccinated, ignoring priority groups. According to a participant, “*The challenge would be having purchased that small quantity, will they really follow the rules of prioritisation to give it to the people who need it most. Prioritisation should be based purely on science and not just political affiliation or social contact or socio-economic status.”* (Participant 10G).

Stakeholders in Bangladesh believe that disregarding priority groups would lead to congestion at vaccination sites and hinder the smooth operations of vaccination teams. Participant 2 emphasised that “*the first challenge will be the security. We need to ensure the security as there will be a great demand of this vaccine.”* (Participant 2B). In Ghana, stakeholders said that the small physical space in health facilities would also contribute to congestion at vaccination centres. One stakeholder said, “*We have challenges with physical space at health facilities for vaccination operations.”* (Participant 11G).

#### Vaccination Program Monitoring and Evaluation Barriers

Stakeholders from Ghana identified poor telecommunication and internet connectivity as barriers to vaccination program surveillance and data collection. Particularly in the countryside, poor internet connectivity delays the upload of vaccination information using data collection software in the field. Health providers have to move to nearby towns or cities to transmit data, which they do not always do. Consequently, vaccination coverage data at the national level are often inaccurate and a false representation of reality. An interviewee said that “*Areas where the telecommunication is poor, especially internet connectivity, there are problems sending service information, stock levels and data to supervisors and with using technologies.”* (Participant 9G).

Stakeholders in both countries discussed the inadequacy of existing surveillance/information management systems. The countries are taking steps are being taken to upgrade them. In Ghana, stakeholders explained that “*the tracking system for COVID-19 is not synced with the national health database. If we synchronise the data, we could gain deep insight[s] to make good decisions”* (Participant 15G).

According to another interviewee, “*The surmax system was not talking to DHIMS2 [District Health Information Management Systems]. So that means you have to get the surmax [data] and then pull it out and go and enter, but now they are doing the integration”* (Participant 16G).

Stakeholders in Bangladesh reported that the inadequate number of IT trained personnel hinder data monitoring activities. One participant said that “*We need more manpower. So, if we cannot appoint skilled manpower to monitor this kind of technical issue, then obviously problems might arise.”* (Participant 4B).

## Discussion

Our study describes the views of national stakeholders on the challenges and barriers to COVID-19 vaccination LMICs, focusing on Ghana and Bangladesh. These countries have considerable experience immunising their populations through the EPI, but this experience differs substantially from rolling out COVID-19 vaccines. In the COVID-19 case, though morbidity and mortality differ across population sub-groups (e.g., by age), the entire population is vaccine-naïve. In contrast, EPI vaccine delivery can focus on smaller population sub-groups, such as infants. Thus, the COVID-19 vaccination rollout poses additional challenges and, as national stakeholders note, exacerbates challenges that already exist with EPI. Bangladesh and Ghana—the first country to receive COVAX-supplied vaccines—have started vaccinating their populations in February and March, respectively; though, incrementally as they acquire more vaccine doses. That gives us the opportunity to examine issues national stakeholder discussed—how the rollout is fairing and what challenges still lie ahead.

Stakeholders discussed their concern for the initially low availability of vaccine doses in their countries despite the COVAX Facility due to the competition for vaccines among HICs. This concern, to a large extent, has materialised. Studies have emphasised that in addition to COVID-19 vaccination speed, equity both within and across countries is important (27–29). Up until April 4, 2021, more than 658 million doses were administered across 151 countries, and though several low-income (7%) and lower-middle-income (20%) countries have started vaccination, upper-middle-income countries (24%) and HICs (49%) represent a far greater proportion of doses administered (30). Studies discussed that expanding COVID-19 vaccine production capacity in LMICs with international collaboration could resolve the vaccine unavailability problem (15, 31).

Our interviews suggest that governments in LMICs have limited funds to afford purchasing enough vaccines for their population. The COVAX Facility aims to supply about two billion COVID-19 vaccine doses starting from 2021 and equally distributing them until participating countries can protect 20% of their population (32). However, funding, shipment challenges, regulatory approval and countries' preparedness for vaccination may delay COVAX's achieving its target (33). Further, since 80% of the population in qualifying LMICs will not be covered by the COVAX vaccine, additional funds will be required to buy vaccines. In addition to COVAX, the African Union Program has organised purchasing vaccines (34), and countries in South Asia have directly negotiated with the manufacturers. LMICs may have to explore several financing sources to afford enough vaccines for their population.

Stakeholders noted that the addition of another vaccine, funded longer-term through co-financing supported by Gavi, could significantly strain COVID-19 vaccination and vaccination programmes more broadly. Issues related to co-financing vaccines have been noted for several vaccines in the past decade (35, 36). Multilateral organisations, international financial institutions, and rich nations should step forward to support LMICs to mobilise funds to combat COVID-19 (15). Studies need to examine co-funding structures to avoid a collapse of vaccination programmes that become unaffordable to LMICs as they transition to self-finance their vaccination programmes (29, 37).

Our study found that adhering to vaccination priority lists could be challenging in LMICs. Countries have been developing National Deployment and Vaccination Plans to aid preparation for vaccination and overcome initial vaccine dose shortages (38). LMICs have developed their prioritisation guidelines in conjunction with WHO assistance. Ghana, for instance, has chosen to begin by prioritising health workers, persons with underlying medical conditions, people over sixty, government officials, and essential service providers in communities with high infection rates (39). However, complying with the list requires adherence by health providers and vaccine consumers, and stakeholders feared a lack of adherence. The stakeholders did not discuss reasons for the potential shift from priority groups. However, the lack of trained providers and suboptimal vaccination monitoring structures that stakeholders discussed could explain why priority may be ignored.

Stakeholders expected to use existing vaccine delivery and administration systems set up for the EPI, with the expansion to the larger population and for an additional vaccine increasing the burden on distribution infrastructure and delivery. Vaccines the EPI distributes target sub-populations, but the entire population requires COVID-19 vaccination, and therefore, the EPI structure alone is likely insufficient. In India, for instance, overcrowding and long waiting times at a COVID-19 vaccination centre resulted in chaos and increased risk of COVID-19 infection among the elderly and comorbid population (40). Overcrowding at COVID-19 vaccination centres has also occurred in HICs: the UK and Canada, for instance, have used general practitioners, hospitals and a national booking centre invitation followed by online booking to control the turn up at vaccination centres; however, congestion has remained (41). LMICs can learn from the issues encountered by countries already further along in the distribution of COVID-19 vaccines, albeit lack of resources may limit many potential solutions.

Countries need to review the capacity of their existing vaccination structures for scaling up COVID-19 vaccination while setting up a system that offers access and reaches all populations for all required doses. Both Ghana and Bangladesh need to consider further the uniqueness of COVID-19 vaccination: (a) vaccination will require additional logistics and a different delivery approach from routine EPI due to high demand, and (b) vaccines come in multiple doses with a short time interval between doses (range 21 to 42 days) which could be interrupted by vaccine shortage. Further, when vaccination sites are located far from residential areas, travel costs, including vaccine price (if not free), can impede vaccination, resulting in low vaccination coverage. Evidence from a systematic review in LMICs supports the findings that travel costs and long distances to vaccination centres deter parents from visiting their vaccination centres (10). Ghanaian stakeholders identified interventions such as drone delivery and providers camping in remote communities; these interventions could bring vaccination sites closer to people's homes and reduce travel costs. Additionally, stakeholders in Ghana reported several issues with the existing EPI infrastructure. Policymakers should place a greater emphasis to resolve these issues as they build on the EPI.

Health systems will also have difficulty ensuring individuals receive all required doses. They will need to set up systems to track who needs a vaccine and identify and contact those requiring second doses. Moreover, vaccination fatigue and the direct and indirect costs of vaccination were described as vaccine uptake barriers by several stakeholders. Ghanaian stakeholders suggested that fatigue from prior vaccination campaigns will increase the difficulty of achieving significant coverage in the case of a multi-dose vaccine.

People are likely to reject vaccination if they perceive vaccine trials were rushed and vaccines are uncertified. People have raised concerns about the safety and efficacy of previous vaccines (42) and COVID-19 vaccines (43), and the media and social media have propagated them (44). Concerns of a rush in vaccine trials and approval threatened confidence in COVID-19 vaccines in India. The fear of a more transmissible alpha mutant (B1.1.7 mutant) forced India to approve the vaccines Covishield and Covaxin for restricted use while phase three trials were ongoing (45). Policymakers acted in a timely manner when they assured the public that an emergency rollout does not mean vaccine safety and efficacy were compromised and that efficacy data will be published upon completion of phase three and four trials. Recent stoppages of vaccination with the AstraZeneca and Johnson & Johnson vaccines in HICs may further increase hesitancy (46, 47). Stakeholders in this study discussed in-country vaccine certification could help increase confidence in COVID-19 vaccines.

Fear of adverse events following vaccination and rumours and wrong beliefs about the COVID-19 vaccine were also noted by stakeholders as a potential obstacle to vaccination. People have already had poor experiences with some vaccines in the past (48, 49), which has led to vaccine rejection, and they are aware of adverse effects that have occurred during clinical trials of COVID 19 vaccines. Bangladesh has had low vaccination registration initially, likely due to concerns of adverse effects, rumours, and anxieties about vaccines (50). In addition to fear of adverse events, inaccurate information about vaccines and sociocultural/religious factors are among the top three reasons for mistrust in vaccines (51). Stakeholders suggested an awareness-building initiative using community and mass media efforts to manage adverse events and inaccurate information.

Public education campaigns (52) and health provider recommendations (53) have effectively addressed vaccine hesitancy in previous vaccination programmes, but the approach may be less effective for COVID-19, particularly in African countries, for at least two reasons: (a) the public distrusts the centralised COVID-19 response due to the lack of urgency and strictness that characterised governments and public health experts' response at the initial stages of the pandemic (54), and (b) non-involvement of local authorities in the design and campaign for preventive measures (55). The use of community influencers, which stakeholders discuss, may be a promising strategy to increasing trust in COVID-19 vaccines (14). The population tends to trust and accept their views (56). LMICs could leverage communities' trust in influencers such as chiefs, pastors, and Imams to debunk conspiracies, allay fears and increase COVID-19 vaccine uptake.

## Conclusion

This qualitative study examines national-level stakeholders' perspectives on demand- and supply-side barriers to COVID-19 vaccination in LMICs, specifically, considering Ghana and Bangladesh as case studies. The study raises awareness about the uniqueness of COVID-19 vaccination for further consideration by decision makers in emerging economies as they prepare to scale up vaccination. Barriers encountered in previous vaccination—including governments' limited funds to buy vaccines, vaccine shortages, few trained service providers, suboptimal cold-chain systems, fear of adverse events, complacency, and rumours of vaccine inefficacy—remain issues for COVID-19 vaccination as LMICs build on their EPI to deliver COVID-19 vaccines. LMICs have learned a lot from EPI vaccination. However, COVID-19 vaccination poses additional challenges, several of which HICs also face. Finally, LMICs may have difficulty administering COVID-19 vaccines according to generated priority lists. Governments should develop their immunisation systems beyond EPI systems to accommodate the pressure of high demand, including by expanding procurement mechanisms and designing localised community influencer-led education campaigns to allay people's fears and increase COVID-19 vaccine acceptance. The study has limitations. Circumstances surrounding COVID-19 and vaccination rapidly change, and the information was retrieved only over a short segment of time. In addition, poor call and internet connectivity (required due to COVID-19 protocols) interrupted interviews and could have reduced interviewees' level of expressiveness on the issues discussed. Future studies should consider the perspectives of service providers and vaccine receivers on barriers to COVID-19 vaccinations and the strategies to overcome them.

## Data Availability Statement

The original contributions presented in the study are included in the article/supplementary material, further inquiries can be directed to the corresponding author/s.

## Ethics Statement

The studies involving human participants were reviewed and approved by the University of Strathclyde and the Ghana Health Service Ethics Review Committee. The patients/participants provided their written informed consent to participate in this study.

## Author Contributions

All authors contributed to conceptualising the study and provided critical feedback to direct analysis and manuscript writing. AS and ETT collected data. NS and ETT reviewed the literature, analysed data, and contributed to manuscript writing. All authors contributed to the article and approved the submitted version.

## Conflict of Interest

The authors declare that the research was conducted in the absence of any commercial or financial relationships that could be construed as a potential conflict of interest.

## Publisher's Note

All claims expressed in this article are solely those of the authors and do not necessarily represent those of their affiliated organizations, or those of the publisher, the editors and the reviewers. Any product that may be evaluated in this article, or claim that may be made by its manufacturer, is not guaranteed or endorsed by the publisher.
